# Extended flexible sigmoidoscopy using water exchange facilitates a complete colon examination without sedation in participants undergoing average risk colorectal cancer screening: results from a randomized trial

**DOI:** 10.1093/jcag/gwae024

**Published:** 2024-07-27

**Authors:** Adrian Bak, Brent Parker, Rafael Perini, Arshbir Aulakh, Caio Oliveira, Wes Richardson, Peter Hirschkorn, Barry Sullivan, Magda Recsky, Tess Orlando, Felix Leung

**Affiliations:** Kelowna Gastroenterology Associates, Kelowna General Hospital, Kelowna, BC, Canada; Legal Services, Provincial Health Services Authority, Vancouver, BC, Canada; Kelowna Gastroenterology Associates, Kelowna General Hospital, Kelowna, BC, Canada; The University of British Columbia, Southern Medical Program, Kelowna, BC, Canada; Kelowna General Hospital, Kelowna, BC, Canada; The University of British Columbia, Southern Medical Program, Kelowna, BC, Canada; The University of British Columbia, Southern Medical Program, Kelowna, BC, Canada; Kelowna General Hospital, Kelowna, BC, Canada; Kelowna General Hospital, Kelowna, BC, Canada; Kelowna Gastroenterology Associates, Kelowna, BC, Canada; Sepulveda Ambulatory Care Center, VA Greater Los Angeles Healthcare System and David Geffen School of Medicine at UCLA, Los Angeles, California, USA

**Keywords:** screening, flexible sigmoidoscopy, sedation, randomized control trial

## Introduction

Colorectal cancer (CRC) is the second most common cause of cancer-related death in men and the third most common in women.^1^ The incidence of and mortality from CRC increases as the population ages.^1^ The Canadian Task Force on Preventive Health Care published recommendations for screening participants at average risk for the disease in 2016.^1^ Despite these recommendations, FS in screening programs in Canada has been limited.

The organized colon cancer screening program in British Columbia (BCCCSP) offers average-risk BC residents ages 50–74 a faecal immunochemical test (FIT) every 2 years.^2^ Neither flexible sigmoidoscopy nor colonoscopy screening is offered to average-risk participants under this program.

Colonoscopy is the gold standard for CRC screening due to its thorough examination of the colon for adenomas and cancers. Yet, its limited use in public screening programs in Canada is due to the need for sedation, which incurs extra costs, resource and personnel use, and participant risks.

FS offers a limited examination of the sigmoid and left colon and is performed without sedation in many settings. Once the splenic flexure is reached, the FS screening procedure is considered completed. Juul et al. conducted a meta-analysis of 4 large, randomized trials, revealing that FS significantly reduced CRC incidence and CRC-related mortality in the distal colon over a 15-year period.^3^ FS screening misses proximal colon adenomas, leading to undetected growths. Serrated lesions, accounting for 20%–30% of CRC cases and often found in the proximal colon, represent a significant alternate pathway to CRC that FS screening overlooks.^4^ The failure of CRC screening programs to lower incidence and mortality from right-sided CRC is partly due to the ineffectiveness of FIT and FS screenings in detecting serrated lesions.

If procedural improvements make FS screening more comfortable and thorough, it could combine FS and colonoscopy benefits, making scope-based screening more viable for public programs than traditional colonoscopy.^5,6^

New colonoscope techniques, like the water exchange (WE) method, may allow for comprehensive, sedation-free screenings. Traditional colonoscopies use gas insufflation (GAS), often requiring sedation due to discomfort, but WE infuse water for better visibility and comfort, enabling most individuals to undergo colonoscopy without sedation.^7^ To date, minimal work has been completed to systematically assess the feasibility of WE to support unsedated screening examinations.

As an alternative to a sedated colonoscopy or FS, this study evaluates using an unsedated extended flexible sigmoidoscopy (EFS) under either WE or GAS, with carbon dioxide (CO_2_) insufflation, as a screening tool. It aims to assess the extent to which EFS using WE enables a full unsedated colon exam compared to EFS using GAS. This study also considers whether either technique demonstrates superior comfort while conserving adenoma detection accuracy without the costs, equipment and personal needs required for traditional colonoscopy.

## Methods

### Study design and participant population

This randomized control trial was conducted at the Kelowna General Hospital in British Columbia (BC), Canada, between 2017 and 2021. The study was institutionally approved by the Interior Health Research Ethics Board and registered on clinicaltrials.gov (NCT03209349). All coauthors had access to the study data and reviewed and approved the final manuscript.

**Figure 1.**
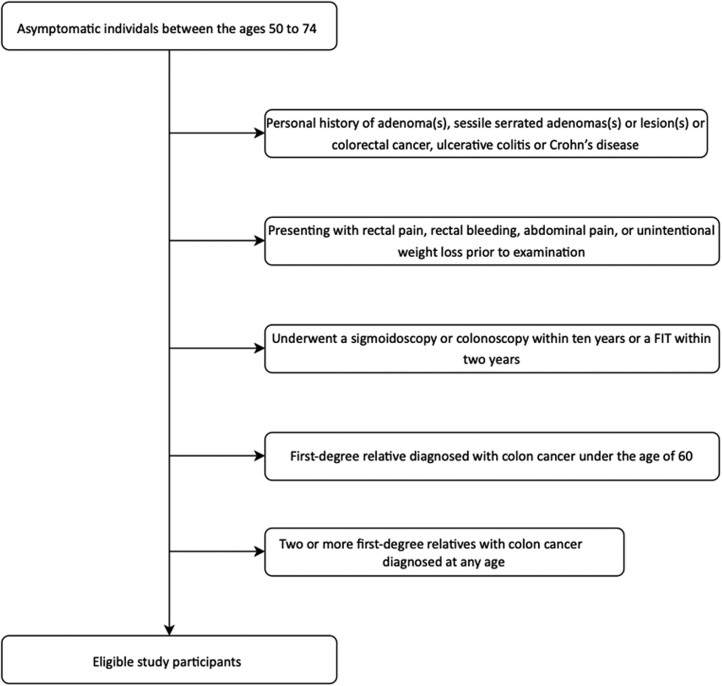
Study criteria.

Local family practice physicians were invited to refer interested average-risk asymptomatic individuals. Interested individuals were screened to confirm they met the study inclusion and exclusion criteria, which were:

- aged 50–74, asymptomatic, no colon-related symptoms or diseases, including rectal pain, rectal bleeding, abdominal pain, or unintentional weight loss;
- no sigmoidoscopy or colonoscopy within 10 years or FIT within 2 years;
- no personal history of adenoma or colon cancer;
- no history of a first-degree relative diagnosed with CRC or multiple adenomas under 60;
- no history of two or more first-degree relatives with CRC at any age;
- no longstanding personal history of inflammatory bowel diseases; and
- no family history of familial adenomatous polyposis or hereditary nonpolyposis CRC (see Figure 1).

These were the criteria that the BCCCSP used at the time of the study to classify a participant as average risk and eligible for screening in the provincial FIT screening program.^2^

Ninety participants were randomized into WE or GAS study arms using a computer tool and secured envelopes, ensuring the performing physician was blinded to the procedure type until the patient was in the procedure room. Nonphysician staff, interviewer, and participants were not informed of the procedure type and were therefore blinded to the procedure type. The endoscope facilitated both GAS and WE, allowing pre-procedure randomization.

### Procedure

Participants ingested a split-dose preparation regimen of standard polyethylene glycol solution (PEG) for bowel preparation. They were advised to take 2 L of PEG the day before the procedure and two litres of PEG 4 to 5 h before the procedure.

Olympus PCF190 series colonoscopes, supporting both WE and GAS, were used for the EFSs. The participating physicians chose between adult or paediatric scopes based on individual preference, and all scopes had variable stiffness available. Two trained gastroenterologists and two colorectal surgeons who routinely performed colonoscopy performed the procedures. Participating physicians had between 5 and 25 years of post-training clinical practice and each physician was in a provincial program that required them to be evaluated on their colonoscopy performance by an independent specialist and to maintain a caseload of over 200 colonoscopies a year. Each physician also had completed a minimum of 50 procedures using WE prior to participation in the study.

In the GAS arm, EFS used minimal CO_2_ insufflation for scope insertion and necessary washing. In the WE arm, water infusion and suction-aided scope insertion with minimal lumen distension, keeping CO_2_ off and aiming for less than 500 cc net water infusion upon cecal intubation as a net infusion of water greater than 500 cc would be more consistent with water immersion another water-based method of colonoscopy, where water is infused without aspiration during the procedure. Air pockets, faeces removal, and infused water suction occurred predominantly during insertion.

Cecal intubation was defined as the passage of the scope’s tip to a point proximal to the ileocecal valve with adequate visualization of the ceacum, the appendiceal orifice, and, in most cases, the terminal ileum. After cecal intubation, CO_2_ was used in all groups for colonic lumen distension and mucosal inspection during withdrawal, when polyp removal occurred. Polyp count and location were recorded.

### Discomfort assessment

Before the procedure, participants were briefed on the Wong–Baker Faces Pain Rating Scale, ranging from 0 (“no hurt”) to 10 (“worst pain imaginable”). A study arm blinded nurse asked the participants to provide their discomfort level during the procedure at scope insertion and every 5 min. If discomfort exceeded 2/10 or participants requested, the scope was withdrawn when safe. Participants reported their recalled maximum discomfort and if the procedure was more uncomfortable than expected both post-procedure and after 24 h by a study arm blinded nurse or study coordinator.

### Participant procedure and pathology and other data

Before the procedure, participants’ demographic data was collected. The study nurse recorded water volumes, procedure length, position changes, diverticulosis rate, and caecum intubation.

Post-procedure pathology reports determined the adenoma detection rate (ADR), sessile serrated adenoma detection rate (SSADR), and serrated lesion detection rate (SLDR). ADR was defined as the proportion of individuals with at least 1 adenoma of any size. SSADR was defined as the proportion of individuals with at least 1 sessile serrated adenoma of any size. SLDR was defined as the proportion of individuals with at least 1 serrated lesion, including any hyperplastic polyps, sessile serrated adenomas without dysplasia, and traditional serrated adenomas with dysplasia. A lesion was considered advanced if the most significant dimension measured more than 10 mm.

### Outcomes

The primary outcome was the cecal intubation rate (CIR). The secondary outcomes were the ADR and the participants’ reported pain immediately after the procedure and recalled pain at approximately 24 h following the procedure. In addition, other metrics, including SSADR, SLDR, participant satisfaction, the willingness to receive the procedure again, position changes, and the amount of water-infused and suctioned insertion and withdrawal, were also recorded.

### Statistical analysis

The initial target study sample size was 100 participants in each arm based on the estimate that CIR achievable by GAS EFS would be near 70%, and a clinically significant difference in CIR would be 20% with a power of 80% and α value of .1.

The anticipated GAS EFS CIR was based on a 2006 study that reported an EFS CIR of 71%.^8^ The threshold difference of 20% or greater was considered clinically significant as it would effectively transform the standards of endoscopic examination from 1 that examines the distal colon and sometimes the full colon to 1 that standardly examines the entire colon.

However, as the study progressed, we became aware that the overall CIR rate was significantly higher than initially anticipated. This observation prompted an interim analysis of 90 participants, which prompted the closing of the study due to the higher than anticipated CIR in both arms, as it was clear that continuation of the study would not demonstrate a clinically significant difference in the high rate of CIR between study arms.

Demographic, clinical, and outcome data between study arms were compared using univariate statistical tests. *P* values < .05 were considered significant. Demographic and clinical data were presented as percentages, counts, and means for continuous data and as absolute and relative frequencies for categorical data. The *χ*^2^ test and Fisher exact test were used to compare categorical data, while the *t-*test was used to compare continuous data.

## Results

### Participant demographics and baseline clinical characteristics

Ninety average-risk, asymptomatic participants were recruited for the study. Due to data mislabelling, two were excluded. The remaining were randomized: 45 to GAS and 43 to WE. Several incomplete questionnaires and the unavailability of 24-h follow-up led to data exclusions, accounting for denominational differences in reported results.

The mean age of participants in the GAS group was 57 years, and 58 years in the WE group. There were no significant differences between age, sex, education level, ethnicity, and marital status across the study arms (Table 1). There was no statistically significant difference in observed diverticulosis rates or Boston Bowl Preparation Scale Scores across study arms (Table 2).

**Table 1.** Characteristics of participants.

Characteristics	Gas insufflation	Water exchange	*P*-value
	*N* (%)	*N* (%)	
Age (years), mean	57	58	.936
Sex (*N*)	45	43	
Female	21 (47%)	23 (53%)	.670
Male	24 (53%)	20 (47%)	
Education (*N*)	35	34	.819
Elementary school or less	0 (0%)	0 (0%)	
Some high school	0 (0%)	1 (3%)	
High School Graduate	7 (20%)	6 (18)	
Some College or University	10 (29%)	11 (32%)	
College or University Graduate	15 (43%)	14 (41%)	
Graduate level degree	3 (9%)	2 (6%)	
Ethnicity (*N*)	34	33	.314
Non-Caucasian	0 (0%)	1 (3%)	
Caucasian/White	34 (100%)	32 (97%)	
Marital status (*N*)	35	34	.57
Married or living as married	27 (77%)	30 (88%)	
Widowed	1 (3%)	0 (0%)	
Never Married	3 (9%)	2 (6%)	
Divorced or separated; not remarried	4 (11%)	2 (6%)	
History of abdominal surgery (*N*)	45	41	.219
Yes	14 (31%)	19 (46%)	

**Table 2.** Boston bowel prep score (by segment and total).

Left colon	Gas insufflation	Water exchange	*P*-value
1	1 (2%)	0 (0%)	
2	4 (9%)	4 (10%)	
3	40 (89%)	37 (90%)	
Subtotal (*N*)	45	41	.904
Transverse colon	Gas insufflation	Water exchange	*P*-value
1	1 (2%)	0 (0%)	
2	9 (20%)	6 (15%)	
3	34 (77%)	35 (85%)	
Subtotal (*N*)	44	41	.509
Right colon	Gas insufflation	Water exchange	*P*-value
1	2 (5%)	0 (0%)	
2	7 (17%)	9 (17%)	
3	33 (79%)	30 (77%)	
Subtotal (*N*)	42	39	.968
Total BBPS score	Gas insufflation	Water exchange	*P*-value
≤5	2 (5%)	0 (0%)	
6	2 (5%)	4 (10%)	
7	3 (7%)	1 (3%)	
8	6 (14%)	5 (13%)	
9	29 (69%)	29 (74%)	
Total (*N*)	42	39	.667

### Participant experience

Table 3 reports participant experience. Discomfort immediately after the procedure was similar. All participants reported maximum recalled pain levels of two or less on a 10-point scale. This overall trend remained similar 24 h, with several individuals reporting a recalled pain level of 3 or more.

**Table 3.** Participant experience metrics.

Characteristics	Gas insufflation	Water exchange		Gas insufflation	Water exchange	
	*N* (%)	*N* (%)	*P*-value	*N* (%)	*N* (%)	*P*-value
Discomfort score	Recalled at discharge			Recalled at 24 h post discharge		
2 or less	45 (100%)	40 (100%)	1	41 (98%)	35 (95%)	.597
3 or more	0 (0%)	0 (0%)		1 (2%)	2 (5%)	
Participant reported satisfaction	Opinion at discharge			Opinion at 24 h post discharge		
9 or 10 (high satisfaction)	34 (77%)	38 (95%)	.012	32 (86%)	34 (81%)	.558
8 or less	10 (23%)	2 (5%)		5 (14%)	8 (19%)	
Scope more uncomfortable than expected	Opinion at discharge			Opinion at 24 h post discharge		
Yes	12 (27%)	7 (18%)	.435	11 (26%)	6 (16%)	.411
No	33 (73%)	33 (83%)		31 (74%)	31 (84%)	
Willing to have the procedure again at next screening interval	Opinion at discharge			Opinion at 24 h post discharge		
Yes	37 (84%)	37 (95%)	.163	37 (88%)	33 (89%)	1
No	7 (16%)	2 (5%)		5 (12%)	4 (11%)	

95% (*N* = 38/40) in the WE group reported a 9 or 10 satisfaction rating on a 10-point scale immediately after the procedure, compared to 77% (*N* = 34/44) in the GAS group (*P* = .012). It diminished slightly at 24 h following the procedure, with 86% and 79% of WE and GAS participants reporting a satisfaction score of 9 or 10.

Immediately after the procedure, there was a non-statistically significant trend toward a higher percentage in the WE group indicating that they would be willing to have the scope again at their next screening interval (*N* = 37/39, 95% in WE, vs. *N*, = 37/44, 84% in GAS, *P* = .163). This trend did not remain at 24 h post-discharge (89% in WE vs. 88% in GAS).

### Clinical outcomes

Table 4 highlights the clinical outcomes. CIR between study arms was similar; 93% (*N* = 38/41) of participants undergoing WE had successful cecal intubation, compared to 91% (*N* = 41/45) in the GAS group (*P* = 0.710). Of those who did not receive an exam to the level of the caecum, 3 GAS participants and two WE participants did not tolerate the examination to the point of the splenic flexure and were considered a failed EFS. In each instance, the failed EFS was due to patient discomfort. Two of these had follow-up colonoscopies; 1 had no polyps detected, and the other had a tubular adenoma detected in the distal transverse colon.

**Table 4.** Comparison of clinical outcomes.

Characteristics	Gas insufflation	Water exchange	
	*N*/total (%)	*N*/total (%)	*P*-value
Clinical outcomes			
Cecal intubation rate	41/45 (91%)	38/41 (93%)	.710
Position changes	13/45 (29%)	8/41 (20%)	.206
Need to apply abdominal pressure during the exam	9/45 (20%)	1/41 (2%)	**.016**
Pathological outcomes			
*N* of adenomas	28	25	
ADR	15/44 (34%)	17/43 (40%)	.660
Advanced ADR (>10 mm)	3/44 (7%)	0/43 (0%)	.241
Proximal ADR	11/44(25%)	12/43(28%)	.811
*N* of sessile serrated adenomas	14	10	
SSADR	6/44 (14%)	9/43 (21%)	.408
Advanced SSADR (>10 mm)	1/44 (2%)	3/43 (7%)	.360
Proximal SSADR	5/44 (11%)	8/43 (19%)	.383
*N* of serrated lesions	30	36	
SLDR	16/44 (36%)	18/43 (42%)	.528
Advanced SLDR (>10 mm)	2/44 (5%)	3/43 (7%)	.676
Proximal SLDR	10/44 (23%)	12/43 (28%)	.628

29% (*N* = 13/45) in the GAS group needed their position changed during the procedure compared to 20% (*N* = 8/41) in the WE group (*P* = .330). There was a statistically significant difference in the need to use abdominal pressure to facilitate scope navigation (*N* = 9/45 (20%) in GAS vs. 1/41 in WE (2%), *P* = .016). The mean procedure time from insertion to withdrawal was 22 min in the GAS and 20 min in the WE. No reported adverse events or complications were associated with the procedures in either arm.

### Pathology

ADR was higher in WE compared to GAS. ADR was 34% in GAS (*N* = 15/44) and 40% in WE (*N* = 17/43) (*P* = .660) (Table 4). Three advanced adenomas (>10 mm in size) were identified in GAS and none in WE. The proximal ADR was 23% (10/44) and 11/43 (26%) (*P* = .811) in the GAS and WE groups.

SSADR was slightly higher in WE than in GAS. SSADR was 14% in GAS (*N* = 6/44) and 21% in the WE group (*N* = 9/43) (*P* = .408) (Table 4). One advanced adenoma (>10 mm in size) was identified in GAS and 3 in WE. The proximal SSADR was 14% (6/44) and 19% (8/43) (*P* = .383) in the GAS and WE.

SLDR was slightly higher in WE than in GAS. SLDR was 36% in GAS (*N* = 16/44), and 42% in WE (*N* = 18/43) (*P* = .528). Three advanced serrated lesions (ASLDRs) were identified in WE and two ASLDRs in GAS. The proximal SLDR was 23% (10/44) and 28% (12/43) (*P* = .628) in the GAS and WE.

## Discussion

Our study’s results show that WE was significantly superior to GAS in patient-reported satisfaction immediately following the procedure. There was a higher but non-statistically significant difference in the rate of cecal intubation and pathology outcomes in the WE group.

Overall, the findings are relevant in considering opportunities to improve CRC screening.

In our study, family practice physicians identified and referred eligible participants, mirroring a potential provincial screening program approach. Some individuals, likely uninterested in CRC screening or preferring FIT screening, were not referred.

Non-sedated EFS is not without any discomfort, and participant expectations and experiences are essential, as they might affect future screening adherence. Sedation is common in traditional colonoscopy, potentially leading to anticipated pain and lower CRC screening participation due to fear of discomfort.^9^ In our study, participant expectations, experience, and willingness to receive the procedure again at their next screening interval were favourable, especially in the WE group.

Cardiopulmonary complications following sedation may occur in up to 1.6% of sedated procedures, which is why adequate monitoring, equipment, and personnel are required during a sedated colonoscopy. These monitoring requirements add to the cost of performing these procedures.^10^ These costs, equipment, personnel needs, and risks associated with traditional colonoscopy screening are avoided in EFS.

CIR is a key quality indicator positively associated with adenoma detection rates.^11^ Current practical guidelines in institutions, like the National Health Service Bowel Cancer Screening Programme and the European Society of Gastrointestinal Endoscopy, expect at least 90% CIR.^12^ Our study found that non-sedated EFS with GAS and WE achieved over 90% CIR, indicating that modern EFS, particularly with WE and a split-dose high-volume preparation, can meet CIR targets. Despite expecting lower CIR with GAS due to discomfort, both techniques generally allowed for a complete, tolerable exam which could improve patient participation if offered endoscopic average-risk CRC screening. We provide, as Table 5, data on volumes of water used by the study arm.

**Table 5.** Volume of water infused.

Volume of water infused (arrival to the caecum)^a^				
	Gas insufflation		Water exchange	
Volume (mL)	Total	% Total	Total	% Total
0–100	6	13	0	0
101–200	3	7	2	5
201–300	16	36	4	10
301–400	9	20	5	12
401–500	6	13	12	29
501+	5	11	18	44
Total	45	100	41	100

Volume of water in suction bottle (arrival to caecum)^a^				
	Gas insufflation		Water exchange	
Volume	Total	% total	Total	% total
0–100	5	11	1	2
101–200	14	31	8	20
201–300	10	22	10	24
301–400	7	16	10	24
401–500	4	9	6	15
501+	5	11	6	15
Total	45	100	41	100
Net volume infused on cecal intubation^a^				

(Volume infused minus volume in suction bottle upon arrival to caecum^a^)				
	Gas insufflation		Water exchange	
Response	Number	% total	Number	% total
Negative-100	33	73	16	39
101–200	8	18	9	22
201–300	2	4	10	24
301–400	2	4	4	10
401–500	0	0	2	5
501+	0	0	0	0
Total	45	100	41	100

^a^Or furthest point of scope advancement.

Another key quality measure is the ADR, reflecting an endoscopist’s success in identifying at least 1 adenomatous polyp. A higher ADR is linked to a reduced risk of interval CRC. A study by Corley et al. found a 1% ADR increase had a 3% decrease in interval CRC.^13^ The current benchmark recommends an overall ADR of 25% for colonoscopy screening in asymptomatic average-risk individuals.^14^ In our study, non-sedated EFS provides an ADR above the recommended benchmark (34% in GAS and 40% in WE). We also consider our results with the other recent studies. A retrospective study by Moreno calculated an overall ADR of 29.7% in 620 patients undergoing screening colonoscopies under sedation, while a different study by Anderson et al. measured an ADR of 29%.^15,16^ As for FS, Cross et al. calculated an overall ADR of 12% using data from the UK Flexible Sigmoidoscopy Screening trial.^17^ The low ADR in FS may be attributed to the limited endoscopic exploration of the proximal colon. A meta-analysis by Cadoni et al. reported a higher ADR and CIR in patients examined with WE compared to GAS.^18^ Cadoni et al. reported a CIR of 97.5% for WE; however, it included colonoscopies using on-demand sedation and full sedation, which, along with the difference in study populations and exclusion criteria, may explain the difference between study results.^19^

Serrated lesions represent a separate pathway to CRC and tend to be in the proximal colon and can be challenging to detect endoscopically because most are very flat. FIT testing for serrated lesions is insensitive.^19^ Preliminary results from the NordICC trial reviewed by Lieberman (2016) reported a serrated lesion detection rate (SLDR) of 8.6% in colonoscopy screening, which likely represents current colonoscopy practices.^4^ The inability to detect serrated lesions contributes to the failure of CRC screening programs to reduce incidence and mortality from CRC on the right side of the colon. Juul et al. ’s findings further support this notion, as they observed no significant reduction in proximal CRC incidence compared to the distal colon with FS.^3^ Our SLDR of 22.7% for GAS and 27.9% for WE demonstrate an opportunity to address this separate pathway to CRC.

This study’s strengths lie in its exclusive focus on a screening population, randomized participant assignments, and blinding of participants and non-endoscopist staff. It captured participant experiences during and 24 h post-procedure, offering a comprehensive view. Four high-volume endoscopists conducted procedures, though further studies are needed to assess generalizability. A limitation was its single-region BC focus and participant over-representation of Caucasians and highly educated individuals, which may affect broader applicability.

Our results demonstrate that both GAS and WE-assisted non-sedated EFS may be a feasible screening test for average-risk individuals. The trend toward superior performance in the WE group and supporting evidence from other WE studies suggest that WE non-sedated EFS may further optimize this screening approach. Further extensive evaluation of its acceptability, performance across different endoscopists, and economic comparison with standard colonoscopy or FIT is warranted.

## Supplementary material

Supplementary material is available at Journal of the *Canadian Association of Gastroenterology* online.

gwae024_suppl_Supplementary_material

## Data Availability

Deidentified data may be available for 5 years post-publication upon request to mrbrentparker@gmail.com, subject to the authors’ data policies, an information sharing agreement, and potential processing fees.

## Notes

ClinicalTrials.gov, Number: NCT03209349
